# When cultural meanings defy expectations: cultural incongruence, epistemic curiosity, critical reflection, and cultural perspective taking in museum education

**DOI:** 10.3389/fpsyg.2026.1942537

**Published:** 2026-09-11

**Authors:** Ganming Yan, Sizhe Wei, Zeheng Cao

**Affiliations:** 1 Digital Media Lab, Wuhan Donghu University, Wuhan, China; 2 Department of Spatial Culture Design, Graduate School of Techno Design, Kookmin University, Seoul, Republic of Korea

**Keywords:** critical reflection, cultural incongruence, cultural perspective taking, epistemic curiosity, museum education, need for cognition, transformative learning

## Abstract

**Introduction:**

Museums increasingly serve as important settings for cultural learning, yet little is known about whether exhibits whose culturally contextualized meanings contradict visitors’ initial expectations can support deeper understanding. Drawing on transformative learning theory, this research examines theoretically connected relationships within a proposed staged framework linking cultural incongruence, epistemic curiosity, critical reflection, and cultural perspective taking. It also investigates the need for cognition as a boundary condition shaping the relationship between critical reflection and cultural perspective taking.

**Methods:**

Two complementary studies were conducted in China. Study 1 employed a randomized scenario experiment with 165 undergraduate students who viewed a fictional museum exhibit under either a low- or high-cultural-incongruence condition. Independent-samples *t*-tests and PLS-SEM were used to examine the effects of the cultural incongruence manipulation on epistemic curiosity and critical reflection, as well as the mediating role of epistemic curiosity. Study 2 used a critical-incident recall survey with 218 adults who described a specific museum or cultural-exhibition experience. Partial least squares structural equation modeling was used to examine the association between critical reflection and cultural perspective taking and the moderating role of need for cognition.

**Results:**

In Study 1, participants in the high-incongruence condition reported significantly greater epistemic curiosity and critical reflection than those in the low-incongruence condition. A significant positive indirect effect of the cultural incongruence manipulation on critical reflection through epistemic curiosity was also observed. In Study 2, critical reflection was positively associated with cultural perspective taking. The interaction between critical reflection and need for cognition was positive and significant, indicating that this association was stronger among participants with higher levels of need for cognition.

**Discussion:**

The findings suggest that expectation-defying cultural meanings do not necessarily impede museum learning. Instead, cultural incongruence may expose limitations in visitors’ existing interpretive frameworks, stimulate inquiry-oriented motivation, and encourage the reconsideration of culturally situated assumptions. By identifying epistemic curiosity as a potential motivational pathway linking the cultural-incongruence manipulation with critical reflection, and by showing a positive association between critical reflection and cultural perspective taking in recalled museum experiences, this research provides complementary evidence for different parts of a proposed transformative-learning framework in museum education.

## 1 Introduction

Museums are gradually evolving from cultural institutions centered on the preservation of collections and the presentation of information into important settings for public learning, cultural understanding, and lifelong education (Heruti and Yahya, 2024; Pantel and Anderson, 2026). Unlike classroom teaching, museum learning generally lacks a uniform learning sequence and explicit assessment requirements. Visitors need to actively construct cultural meanings by drawing on exhibits, interpretive texts, and their experiences (Kim et al., 2024; Li, 2024; King et al., 2025). During this process, the cultural meaning of an exhibit cannot always be directly inferred from its appearance. A brightly colored object may be used in a solemn farewell ritual, while a symbol that appears celebratory may convey loss, restraint, or taboo. Such exhibits may create a conflict between visitors’ initial cultural judgments and the contextual information they subsequently receive, thereby forming a noteworthy situation of expectancy violation in museum learning. This expectation-defying encounter raises an important question for museum education: how do visitors respond when an initially plausible cultural interpretation is disrupted by subsequently provided cultural information?

Existing research on museum learning has extensively examined how exhibits promote learning engagement, historical understanding, and intercultural communication through information presentation, cultural narratives, and sensory experiences (Erbaş et al., 2026; Kim et al., 2024; Li, 2024). However, the relationship between exhibit meanings and visitors’ existing cultural expectations has not received sufficient attention. In particular, cultural incongruence differs from general novelty or cultural difference. It refers to the inconsistency between the initial cultural judgment that visitors form on the basis of an exhibit’s observable features and the actual cultural meaning revealed by contextual information obtained later. Existing research has not yet systematically explained whether this type of expectancy violation can serve as an educationally valuable feature of an exhibit or through what psychological processes visitors respond to it.

To explain how visitors respond to such expectancy violations, it is necessary to identify the psychological response that may turn an explanatory discrepancy into deeper engagement with cultural meaning. In this regard, research on epistemic emotions provides an important perspective for understanding this initial response. Uncertainty, contradiction, and insufficient explanation can elicit curiosity, surprise, or confusion and shape subsequent information seeking and engagement in learning (Berweger et al., 2023; Jheng et al., 2025; Noordewier and Gocłowska, 2024). Among these responses, epistemic curiosity reflects an individual’s desire to seek further information and understanding upon recognizing gaps in knowledge or explanation (Erdemli et al., 2025). However, existing research has paid limited attention to the epistemic responses triggered when cultural meanings violate expectations, and it has not fully explained whether this inquiry-oriented motivation can further prompt visitors to reconsider their initial cultural judgments. At the same time, research on critical reflection emphasizes the re-evaluation of existing assumptions, experiences, and ways of interpreting information (O’Flaherty et al., 2025; Tight, 2024), yet its emotional and motivational antecedents have not been systematically examined in museum-based cultural learning. Accordingly, how epistemic curiosity elicited by cultural incongruence is related to critical reflection represents a key issue in understanding the educational role of expectancy violation.

Yet critical reflection is not the endpoint of museum-based cultural learning. Its educational significance also depends on whether reconsidering one’s initial cultural judgments helps visitors move beyond familiar standards of interpretation and understand exhibits from the standpoint of another culture. Research on intercultural education suggests that cultural education should not stop at identifying differences between cultures, but should also help learners understand the values, beliefs, and historical contexts underlying cultural practices (Conti, 2025; Erbaş et al., 2026). Cultural perspective taking reflects this more context-sensitive form of cultural understanding by encouraging visitors to interpret others’ viewpoints, emotions, and behaviors from a particular cultural or historical context. However, existing museum research has more often discussed it as an educational strategy or learning outcome, while paying relatively limited attention to how it develops and through what psychological mechanisms (Savenije and De Bruijn, 2017; Sherman et al., 2020). Importantly, however, recognizing the limitations of one’s initial cultural judgment does not automatically lead to the construction of an alternative cultural interpretation. Doing so may require additional cognitive effort to consider unfamiliar values, historical contexts, and culturally situated meanings. Need for cognition reflects a stable tendency to engage in and enjoy complex thinking and is closely related to information processing, cognitive engagement, and learning performance (Liu and Nesbit, 2024). It is therefore necessary to further examine when critical reflection is more likely to be associated with cultural perspective taking.

These gaps leave unresolved how expectation-defying cultural encounters are related to deeper forms of museum-based cultural learning and what psychological and individual factors may help explain these relationships. Accordingly, the purpose of this research is to examine theoretically connected relationships among cultural incongruence, epistemic curiosity, critical reflection, cultural perspective taking, and need for cognition within the overarching framework of transformative learning theory. Specifically, this study addresses the following research questions:

*RQ1*: How does cultural incongruence influence visitors’ epistemic curiosity and critical reflection in museum-based cultural learning?

*RQ2*: What role does epistemic curiosity play in linking cultural incongruence with critical reflection?

*RQ3*: How is critical reflection associated with cultural perspective taking, and to what extent is this association contingent on visitors’ need for cognition?

To address these research questions, two complementary studies examine distinct but theoretically connected parts of the proposed framework. Study 1 uses a two-group randomized scenario experiment to examine the effects of cultural incongruence on epistemic curiosity and critical reflection, as well as the mediating role of epistemic curiosity. Study 2 uses a critical-incident recall survey to examine the relationship between critical reflection and cultural perspective taking and the moderating role of need for cognition.

## 2 Literature review and hypothesis development

### 2.1 Transformative learning in museum education

Transformative learning theory focuses on how individuals reconsider their existing assumptions and develop new perspectives when their established meaning frameworks are challenged (Mezirow, 1978; Tennant, 1993). When new experiences are difficult to accommodate within existing beliefs and ways of interpretation, individuals may become aware of the limitations of their prior understanding and reconstruct meaning through inquiry and critical reflection. Therefore, transformative learning differs from the simple accumulation of factual knowledge, as its core lies in changes to interpretive frameworks and the assumptions on which they are based (Taylor, 2007; McClain, 2024). Recent research has further emphasized that disorienting experiences do not necessarily lead to transformation in themselves. Their effects depend on how learners interpret those experiences and how particular social and learning contexts shape the reflective process (Holdo, 2023; McClain, 2024).

Museums provide an appropriate cultural context for transformative learning. Visitors generally form initial interpretations based on exhibit characteristics and their cultural experiences, while the values, symbols, or practices revealed by exhibits may challenge these existing judgments. Research conducted in art museums has also shown that object observation, discussion, and experiential learning in museums can prompt learners to examine their existing assumptions and develop new ways of understanding (Chisolm et al., 2020; Yoo, 2023). Accordingly, this study views cultural incongruence as a disorienting experience that may challenge existing cultural meaning frameworks, critical reflection as the examination of prior cultural judgments and their underlying assumptions, and cultural perspective taking as a proximal manifestation of this reflective process in museum-based cultural learning. Epistemic curiosity is not an original core concept of transformative learning theory; this study conceptualizes it as an inquiry-oriented motivation through which visitors respond to cultural disruption, thereby supplementing explanations of how disorienting experiences develop into critical reflection. Need for cognition is regarded as an individual condition that influences whether reflection can further extend to the complex processing of cultural perspectives.

### 2.2 Cultural incongruence, epistemic curiosity, and critical reflection

This study defines cultural incongruence as the perceived inconsistency between the initial judgment of cultural meaning that visitors form on the basis of an exhibit’s observable features and the actual cultural meaning revealed by subsequent contextual information. It differs from general novelty or cultural difference. Novelty primarily reflects the extent to which a stimulus is unfamiliar relative to an individual’s prior experience, whereas cultural difference emphasizes distinctions among cultures in values, norms, or practices. By contrast, cultural incongruence means that visitors have already formed an apparently reasonable cultural interpretation, but this interpretation cannot be reconciled with the contextual information obtained later (Meyers-Levy and Tybout, 1989; Kidd and Hayden, 2015). Thus, what visitors encounter is not simply a lack of information, but the failure of an existing interpretive framework within the current cultural context.

From the perspective of transformative learning theory, when new experiences cannot be adequately explained by existing meaning frameworks, individuals may begin to reconsider their prior judgments and the assumptions underlying them (Taylor, 2007; Holdo, 2023). Museum visitors typically construct the meanings of exhibits based on prior knowledge, symbolic associations, and lived experience, and these interpretive foundations often operate without being consciously examined (Mezirow, 1998; O’Connor et al., 2020). When the actual cultural function or symbolic meaning of an exhibit violates visitors’ initial expectations, what they encounter is not simply new information, but a contradiction between their prior interpretation and the contextual information provided later. This interpretive failure may lead visitors to recognize that their initial understanding of the exhibit was shaped by their existing cultural experience and may not necessarily apply to other cultural contexts. Critical reflection involves examining the premises and assumptions on which judgments are based, as well as the boundaries of their applicability, rather than merely replacing prior understanding with new knowledge (Mezirow, 1998; Kember et al., 2000; O’Flaherty et al., 2025). Therefore, the more salient the cultural incongruence, the more likely visitors are to reconsider their prior cultural interpretations and the foundations on which those interpretations were formed. Accordingly, this study proposes:

*H1*: Cultural incongruence is positively associated with critical reflection.

Beyond prompting visitors to reconsider their initial cultural judgments, cultural incongruence may also create a need for further understanding. Critical reflection primarily serves an evaluative function by directing attention toward the assumptions and interpretive frames underlying one’s initial judgment, whereas epistemic curiosity primarily serves an acquisitional function by motivating individuals to seek additional information and explanation when they encounter a gap in understanding (Mezirow, 1998; Kember et al., 2000; Litman et al., 2005; Schmidt and Rotgans, 2021). In this sense, the two responses are distinct: one concerns the re-evaluation of an existing interpretation, while the other concerns the pursuit of information needed to resolve what remains unexplained.

Such an acquisitional response is particularly likely when an exhibit’s actual cultural meaning conflicts with the meaning implied by its appearance. This discrepancy may reveal that visitors’ existing knowledge is insufficient to provide a coherent explanation, thereby creating a need to understand the exhibit’s cultural background, symbolic logic, and social function (Loewenstein, 1994; Vogl et al., 2020). Epistemic curiosity is activated by such perceived gaps in understanding and motivates further information seeking and exploration (Schmidt and Rotgans, 2021; Erdemli et al., 2025). Compared with expectation-consistent cultural information, cultural incongruence should therefore make the need for further explanation more salient and elicit greater epistemic curiosity. Therefore, this study proposes:

*H2*: Cultural incongruence is positively associated with epistemic curiosity.

Epistemic curiosity may also move visitors beyond acquiring new information toward critical reflection. Curiosity-driven inquiry does not merely increase the amount of information obtained; it also prompts individuals to compare their existing interpretations with newly acquired information and to continue asking why the two differ (Litman et al., 2005; Schmidt and Rotgans, 2021). In museum-based cultural learning, visitors may initially focus on the question, “What does this exhibit actually mean?” As their inquiry deepens, their attention may shift to, “Why did I form a different judgment?” This cognitive shift makes it easier for visitors to identify the cultural associations and default assumptions underlying their initial interpretations and to reconsider whether these assumptions apply to the current cultural context. Epistemic curiosity thus sustains the cognitive engagement required to process cultural inconsistency, enabling visitors, through comparison between their initial expectations and the actual cultural meaning, to move gradually from filling an explanatory gap to examining their ways of interpretation. Accordingly, epistemic curiosity elicited by cultural incongruence may constitute an important psychological mechanism through which cultural incongruence promotes critical reflection. Based on this reasoning, this study proposes:

*H3*: Epistemic curiosity mediates the relationship between cultural incongruence and critical reflection.

### 2.3 Critical reflection and cultural perspective taking

Critical reflection may provide an important cognitive foundation for cultural perspective taking. Visitors’ initial understanding of an exhibit is often shaped by their cultural symbols, value standards, and lived experiences, while these interpretive premises are rarely examined consciously. Critical reflection can help individuals recognize that their understanding is neither the only possible interpretation nor universally valid, but is constrained by a particular cultural framework (Mezirow, 1998; Carlson et al., 2025). According to transformative learning theory, re-evaluating existing assumptions can weaken the dominance of established meaning frameworks and create space for alternative understandings (Mezirow, 1998; Taylor, 2007; Holdo, 2023). Therefore, when visitors recognize that their cultural experiences are insufficient to explain the meaning of an exhibit, they may be more likely to consider the values, beliefs, historical background, and social functions of the culture to which the exhibit belongs, and to understand the exhibit from the standpoint of members of that culture. Compared with simply recognizing cultural differences, cultural perspective taking places greater emphasis on temporarily moving beyond one’s standpoint to understand how other cultural groups assign meaning to objects and practices. This is also an important expression of ethnocultural perspective taking (Wang et al., 2003). Accordingly, the deeper the visitors’ critical reflection, the higher their level of cultural perspective taking may be. Therefore, this study proposes:

*H4*: Critical reflection is positively associated with cultural perspective taking.

### 2.4 The moderating role of need for cognition

Although critical reflection can make visitors aware that their prior cultural judgments are constrained by their experiences and assumptions, such awareness does not necessarily translate into cultural perspective taking. The latter requires individuals not only to loosen their reliance on existing interpretive frameworks but also to integrate the historical background, values, beliefs, and social functions of the culture represented by the exhibit, thereby constructing a cultural interpretation that differs from their initial judgment. This process generally involves sustained comparison of multiple sources of information and relatively effortful cognitive processing.

Need for cognition refers to a stable tendency to engage in and enjoy complex thinking (Liu and Nesbit, 2024). Individuals high in need for cognition are generally more willing to invest cognitive effort rather than rely on simple and intuitive explanations (Lins de Holanda Coelho et al., 2020). Existing meta-analytic evidence also shows a stable positive relationship between need for cognition and learning and cognitive performance, suggesting that it can support sustained processing of cognitively demanding information (Liu and Nesbit, 2024). Therefore, when the need for cognition is high, visitors may be more likely, after recognizing the limitations of their prior cultural assumptions, to continue analyzing the cultural background of the exhibit and coordinate their interpretation with the meaning framework of another culture. As a result, critical reflection may be more strongly associated with cultural perspective taking. In contrast, visitors low in need for cognition may be less willing to continue engaging in complex processing even after recognizing the limitations of their initial judgment, and may remain at the level of acknowledging differences or accepting new facts. Therefore, this study proposes:

*H5*: Need for cognition positively moderates the relationship between critical reflection and cultural perspective taking, such that the relationship is stronger at higher levels of need for cognition.

Based on the preceding hypotheses, this study proposes a staged model in which cultural incongruence is associated with critical reflection both directly and indirectly through epistemic curiosity. The model further specifies cultural perspective taking as a proximal outcome of critical reflection and need for cognition as a boundary condition shaping this relationship (see Figure 1).

**Figure 1:**
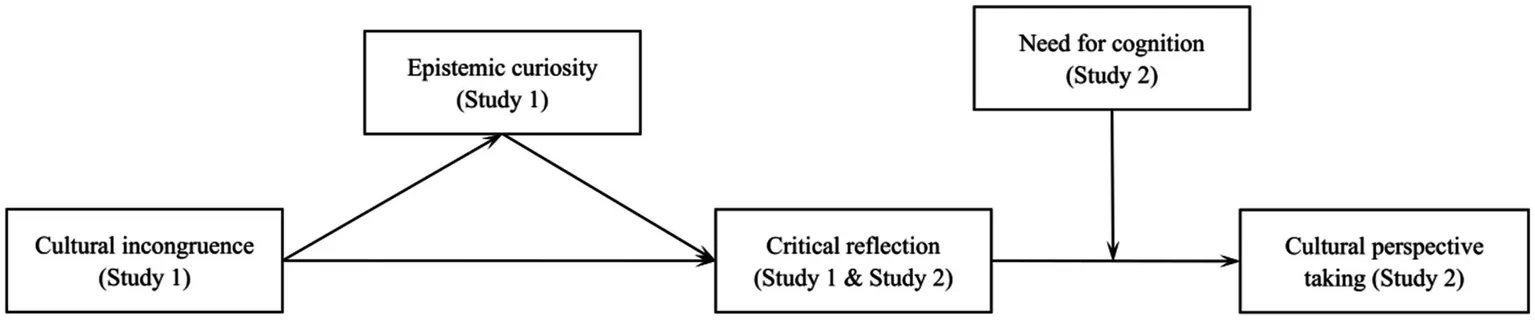
Proposed conceptual framework and relationships examined across Study 1 and Study 2.

## 3 Overview of the study design

To examine different parts of the proposed staged model, this research comprised two complementary studies. Study 1 employed a two-group randomized scenario experiment with undergraduate students recruited online from universities in China. It examined whether cultural incongruence influenced critical reflection directly and indirectly through epistemic curiosity. Study 2 employed an online critical-incident recall survey, with participants recruited through a combination of general online recruitment and purposive sampling. It examined the relationship between critical reflection and cultural perspective taking, as well as the moderating role of need for cognition.

The two studies examined distinct but theoretically connected parts of the conceptual model. Study 1 provided experimental evidence for the immediate effects of cultural incongruence on epistemic curiosity and critical reflection, whereas Study 2 focused on the downstream relationship between critical reflection and cultural perspective taking in participants’ recalled museum or cultural-exhibition experiences. This complementary design allowed the antecedent process to be examined under controlled conditions and the downstream relationship to be evaluated in naturally occurring museum experiences. Because the studies used independent samples, they were designed to examine separate parts of the framework rather than the complete sequence within the same participants or museum experience. Table 1 summarizes the overall research design.

**Table 1:** Overview of the research design.

Study	Design	Core relationships examined	Research context
Study 1	Two-group randomized scenario experiment	Cultural incongruence → epistemic curiosity → critical reflection	Simulated museum exhibit
Study 2	Critical-incident recall survey	Critical reflection × need for cognition → cultural perspective taking	Actual museum or cultural-exhibition experience

## 4 Study 1

### 4.1 Method

#### 4.1.1 Participants and design

Study 1 employed a two-group between-subjects randomized scenario experiment conducted between January and February 2026. Undergraduate students were recruited online with the assistance of student associations at two universities in China. Participants were required to be at least 18 years old, to have visited a museum or cultural exhibition within the previous 12 months, and not to have previously viewed or completed the experiment. A total of 200 students were recruited and randomly assigned to the low- or high-cultural-incongruence condition, with 100 initially recruited for each condition. After data-quality screening, 165 responses were retained, including 87 participants in the low-incongruence condition and 78 in the high-incongruence condition. Participants ranged in age from 18 to 24 years, with a mean age of 19.73 years (*SD* = 1.58). Of the retained sample, 97 participants were women (58.8%) and 68 were men (41.2%).

#### 4.1.2 Stimulus development and procedure

The stimulus was developed to manipulate the incongruence between an exhibit’s visual appearance and its stated cultural meaning. Participants viewed a fictional exhibit, the Sunbird Mask, a brightly colored wooden bird-shaped mask attributed to the fictional Nalo community. In the low-incongruence condition, the mask was described as being used in a harvest celebration and symbolizing joy, good fortune, and abundance, meanings broadly consistent with its bright appearance. In the high-incongruence condition, the same mask was described as being used in a solemn farewell ritual and symbolizing restraint, loss, and temporary separation, thereby creating a clear mismatch between appearance and cultural meaning. The two versions used the same image, title, object information, layout, and comparable text length, while differing in the ritual context and cultural meaning described in the exhibit label. The fictional exhibit and community were used to reduce potential contamination from prior knowledge.

After providing informed consent and completing the eligibility screening, participants were randomly assigned to one of the two conditions. They were asked to imagine visiting a special museum exhibition and to read the exhibit label as if encountering the object in person. Immediately afterward, they completed measures of epistemic curiosity and critical reflection, followed by the manipulation check, comprehension and attention checks, and demographic questions. The manipulation check was intentionally administered after the focal outcome measures to avoid explicitly drawing participants’ attention to the discrepancy between the exhibit’s appearance and its cultural meaning before they reported their epistemic curiosity and critical reflection. This ordering was intended to minimize the possibility that the manipulation-check items themselves would prime the focal responses. Participants were then debriefed and informed that both the exhibit and the Nalo community were fictional.

#### 4.1.3 Measures

All measurement items were adapted from established scales and contextualized to the museum-exhibit scenario while preserving their original conceptual meanings. The English-language items were translated into Chinese and independently back-translated to ensure semantic equivalence. Minor wording adjustments were made to fit the museum-exhibit scenario while preserving the intended meanings of the original items. Unless otherwise stated, all items were measured using 7-point Likert scales. Epistemic curiosity was assessed using six items adapted from Schmidt and Rotgans (2021). A sample item was “I am curious to learn more about the culture behind this exhibit.” Critical reflection was measured with four items adapted from Kember et al. (2000), including “This exhibit made me rethink how I usually interpret cultural objects.” The negatively worded epistemic-curiosity item was reverse-coded. The manipulation check for cultural incongruence used three items adapted from the recognition-of-contradiction dimension developed by Lee et al. (2003). The items were reworded to capture the perceived discrepancy between the exhibit’s appearance and its stated cultural meaning. A sample item was “There was a clear mismatch between how the exhibit looked and what it meant in this culture.”

### 4.2 Results

#### 4.2.1 Manipulation check

An independent-samples *t*-test was conducted to assess the effectiveness of the cultural-incongruence manipulation. Participants in the high-incongruence condition reported significantly greater perceived cultural incongruence (*M* = 5.12, *SD* = 0.95) than those in the low-incongruence condition (*M* = 2.85, *SD* = 0.84), *t*(163) = 16.29, *p* < 0.001. The mean difference was 2.27, with a 95% confidence interval ranging from 1.99 to 2.54. These results confirmed that the experimental manipulation was successful.

#### 4.2.2 Hypothesis testing

Independent-samples *t*-tests were conducted to examine the effects of cultural incongruence on critical reflection and epistemic curiosity. Participants in the high-cultural-incongruence condition reported significantly greater critical reflection (*M* = 3.87, *SD* = 1.33) than those in the low-cultural-incongruence condition (*M* = 3.17, *SD* = 1.30), *t*(163) = 3.39, *p* = 0.001. Participants in the high-cultural-incongruence condition also reported significantly greater epistemic curiosity (*M* = 4.32, *SD* = 1.29) than those in the low-cultural-incongruence condition (*M* = 3.14, *SD* = 1.23), *t*(163) = 6.06, *p* < 0.001. These findings supported H1 and H2, respectively.

Before testing the mediation model, the measurement properties of epistemic curiosity and critical reflection were assessed. Composite reliability (CR) was 0.932 for epistemic curiosity and 0.893 for critical reflection, while the corresponding average variance extracted (AVE) values were 0.694 and 0.679, respectively, indicating satisfactory internal consistency and convergent validity. The HTMT value between epistemic curiosity and critical reflection was 0.482, well below the recommended threshold of 0.85, supporting discriminant validity (Hair et al., 2019).

The mediating effect of epistemic curiosity was then examined using PLS-SEM bootstrapping, with age and gender included as control variables. The experimental condition was dummy-coded as 0 for the low-incongruence condition and 1 for the high-incongruence condition. The indirect effect of the cultural incongruence manipulation on critical reflection through epistemic curiosity was positive and significant (*β* = 0.331, 95% *CI* [0.175, 0.536], *t* = 3.59, *p* < 0.001), supporting H3. In the mediation model, the direct effect of the cultural incongruence manipulation on critical reflection was not significant (*β* = 0.196, 95% *CI* [−0.136, 0.508], *t* = 1.19, *p* = 0.233), indicating that epistemic curiosity fully mediated this relationship. Neither age nor gender had a significant effect on critical reflection.

## 5 Study 2

### 5.1 Method

#### 5.1.1 Participants and procedure

Study 2 employed an online critical-incident recall survey conducted in China between March and May 2026. Participants were recruited through social media platforms, online survey communities, and university- and museum-related interest groups using a combination of general online recruitment and purposive sampling. Eligible participants were required to be at least 18 years old, to have visited a museum or cultural exhibition either in person or online within the previous 12 months, and to be able to clearly recall one specific exhibit or cultural object from that visit. Responses were screened for eligibility, duplicate submissions, failure of the instructed-response attention check, excessively short completion times, and evident straight-lining or patterned responding. After data-quality screening, 218 responses were retained. Participants ranged in age from 18 to 60 years (*M* = 26.94, *SD* = 8.61); 119 were women (54.6%), and 99 were men (45.4%). Regarding educational background, 41 participants (18.8%) reported high school education or below, 67 (30.7%) college-level education, 82 (37.6%) bachelor’s-level education, and 28 (12.8%) postgraduate education.

After providing informed consent and completing the eligibility screening, participants were asked to recall one specific exhibit or cultural object that they remembered clearly from a museum or cultural exhibition visited during the previous 12 months. Rather than evaluating the museum or exhibition as a whole, they were instructed to focus on an object that communicated a particular cultural belief, value, practice, tradition, or way of life. This focus on a specific object was intended to provide a concrete experiential reference for participants’ subsequent responses, while allowing the recalled exhibits and cultural contexts to reflect the diversity of their actual museum experiences. To anchor the recalled experience, participants briefly identified the exhibit or object, the culture or tradition it represented, and the cultural meaning or use described by the exhibition label. They then completed measures of critical reflection, need for cognition, and cultural perspective taking with reference to the same exhibit, followed by an attention check and demographic questions. Participation was voluntary and anonymous.

#### 5.1.2 Measures

All measurement items were drawn from established scales and contextualized to the museum-exhibit setting while preserving their original conceptual meanings. The English items were translated into Chinese and independently back-translated to ensure semantic equivalence. Minor wording adjustments were made so that participants answered with reference to the same exhibit or cultural object identified in the recall task. Unless otherwise stated, all constructs were measured using 7-point Likert scales. Critical reflection was measured using the same 4-item scale employed in Study 1.

Need for cognition was assessed using the 6-item short form developed by Lins de Holanda Coelho et al. (2020), which captures individuals’ general tendency to engage in and enjoy effortful thinking. A sample item was “I prefer complex problems to simple ones.” Cultural perspective taking was measured using 7 items adapted from Wang et al. (2003) and contextualized to assess the extent to which participants attempted to understand the recalled exhibit from the cultural standpoint represented in the exhibition. A sample item was “I tried to understand this exhibit within its cultural context rather than judging it only by my usual standards.” Age, gender, and educational level were included as control variables.

### 5.2 Results

#### 5.2.1 Common method bias (CMB) assessment

To evaluate whether the use of self-reported data introduced substantial CMB, two complementary diagnostic procedures were performed. The unrotated exploratory factor analysis yielded three factors with eigenvalues greater than 1, with the first factor accounting for 33.54% of the total variance, well below the conventional 50% threshold. The common method factor analysis further showed (Table 2) that the substantive factor loadings were all statistically significant and explained an average of 75.3% of the indicator variance, whereas the method factor accounted for only 1.1% on average; moreover, only one method-factor loading was significant. These diagnostic results indicated that CMB was unlikely to pose a serious threat to the interpretation of the findings (Podsakoff et al., 2003; Liang et al., 2007).

**Table 2:** Common method factor analysis.

Construct	Indicator	Substantive factor loading (*R*_1_)	*R*_1_^2^	Method factor loading (*R*_2_)	*R*_2_^2^
Cultural perspective taking (CPT)	CPT1	0.837^***^	0.701	0.029	0.001
	CPT2	0.771^***^	0.594	−0.228	0.052
	CPT3	0.842^***^	0.709	−0.188	0.035
	CPT4	0.912^***^	0.832	−0.037	0.001
	CPT5	0.818^***^	0.669	0.063	0.004
	CPT6	0.792^***^	0.627	0.270^*^	0.073
	CPT7	0.823^***^	0.677	0.095	0.009
Critical reflection (CR)	CR1	0.902^***^	0.814	0.018	0.000
	CR2	0.893^***^	0.797	0.071	0.005
	CR3	0.872^***^	0.760	−0.095	0.009
	CR4	0.834^***^	0.696	0.002	0.000
Need for cognition (NC)	NC1	0.895^***^	0.801	−0.033	0.001
	NC2	0.911^***^	0.830	−0.034	0.001
	NC3	0.905^***^	0.819	0.029	0.001
	NC4	0.911^***^	0.830	−0.017	0.000
	NC5	0.902^***^	0.814	0.044	0.002
	NC6	0.911^***^	0.830	0.009	0.000
Average		0.867	0.753	0.000	0.011

^***^p < 0.001, ^*^p < 0.05.

#### 5.2.2 Measurement model evaluation

As reported in Table 3, all indicator loadings ranged from 0.748 to 0.925 and exceeded the recommended threshold of 0.70. Cronbach’s *α* values ranged from 0.898 to 0.956, while CR values ranged from 0.929 to 0.964, indicating satisfactory internal consistency. The AVE values were 0.686 for cultural perspective taking, 0.766 for critical reflection, and 0.816 for need for cognition, all above the recommended threshold of 0.50 and therefore supporting convergent validity (Hair et al., 2019).

**Table 3:** Measurement model results.

Construct	Item	Cronbach’s *α*	CR	AVE	Loading
Cultural perspective taking (CPT)	CPT1	0.923	0.938	0.686	0.831
	CPT2				0.748
	CPT3				0.825
	CPT4				0.914
	CPT5				0.826
	CPT6				0.813
	CPT7				0.831
Critical reflection (CR)	CR1	0.898	0.929	0.766	0.904
	CR2				0.899
	CR3				0.862
	CR4				0.835
Need for cognition (NC)	NC1	0.956	0.964	0.816	0.870
	NC2				0.896
	NC3				0.903
	NC4				0.903
	NC5				0.925
	NC6				0.921

The distributions of the focal constructs were also examined. The absolute skewness values ranged from 0.055 to 0.245, and the absolute kurtosis values ranged from 1.235 to 1.678, indicating no serious departure from normality. As shown in Table 4, the square root of the AVE for each construct was 0.828 for cultural perspective taking, 0.875 for critical reflection, and 0.903 for need for cognition, and each value exceeded the corresponding inter-construct correlations. The HTMT ratios ranged from 0.106 to 0.418, remaining well below the conservative threshold of 0.85. These results supported the reliability, convergent validity, and discriminant validity of the measurement model (Hair et al., 2019).

**Table 4:** Descriptive statistics and discriminant validity results.

Construct	*M*	*SD*	Skewness	Kurtosis	1	2	3
Cultural perspective taking	3.608	1.305	0.245	−1.235	**0.828**	0.418	0.106
Critical reflection	3.770	1.486	−0.055	−1.279	0.390	**0.875**	0.155
Need for cognition	3.588	1.778	0.113	−1.678	0.113	−0.134	**0.903**

Bold diagonal values represent the square roots of AVE; values below the diagonal represent correlations; values above the diagonal represent HTMT ratios.

#### 5.2.3 Hypothesis testing

The estimated model yielded an SRMR of 0.050, below the commonly used guideline of 0.08. The model explained 27.7% of the variance in cultural perspective taking (*R*^2^ = 0.277), and the *Q*^2^ value of 0.184 indicated satisfactory predictive relevance. The VIF values for all predictors ranged from 1.005 to 1.051, remaining well below the recommended threshold and indicating that multicollinearity was not a concern.

As shown in Table 5, critical reflection was significantly and positively associated with cultural perspective taking (*β* = 0.362, 95% CI [0.244, 0.479], *p* < 0.001), supporting H4. The interaction between critical reflection and need for cognition was also positive and significant (*β* = 0.309, 95% CI [0.156, 0.411], *p* < 0.001), supporting H5 and indicating that the relationship between critical reflection and cultural perspective taking was stronger at higher levels of need for cognition. The control effects of age, gender, and educational level were all nonsignificant.

**Table 5:** Results of hypothesis testing.

Path	*β*	LLCI	ULCI	*p* value	VIF	*R* ^2^	*Q* ^2^	Support
Panel A. Tests of the study hypotheses								
Critical reflection → Cultural perspective taking	0.362	0.244	0.479	<0.001	1.046			Yes
Critical reflection × Need for cognition → Cultural perspective taking	0.309	0.156	0.411	<0.001	1.043	0.277	0.184	Yes
Panel B. Effects of the control variables								
Age → Cultural perspective taking	−0.038	−0.163	0.089	0.560	1.013			/
Gender → Cultural perspective taking	0.091	−0.149	0.336	0.458	1.005			/
Educational level → Cultural perspective taking	−0.040	−0.152	0.085	0.513	1.051			/

*R*^2^ and *Q*^2^ values refer to the corresponding endogenous constructs rather than to individual structural paths.

## 6 General discussion

### 6.1 Discussion of results

The findings of two studies are consistent with different parts of a proposed staged account of cultural learning in museum settings. Study 1 showed that participants reported greater epistemic curiosity and critical reflection when an exhibit’s actual cultural meaning was inconsistent with the initial interpretation suggested by its appearance. This finding indicates that expectation-defying cultural meanings do not necessarily impede understanding; they may also make visitors aware that their existing interpretations are insufficient to explain an exhibit’s cultural function. The defining feature of cultural incongruence is not simply unfamiliarity, but the failure of an initially plausible interpretation after additional contextual information is provided. The resulting explanatory tension makes gaps in knowledge more salient and encourages visitors to reconsider the cultural experiences underlying their initial judgments. This pattern is consistent with research showing that expectancy violations, misconceptions, and unresolved explanatory problems can elicit epistemic curiosity and promote knowledge exploration (Vogl et al., 2020; Berweger et al., 2023). It also accords with transformative learning research, suggesting that learners may reassess established meaning frameworks when new experiences cannot be adequately accommodated within them (Taylor, 2007; Holdo, 2023). Therefore, the findings are consistent with the possibility that cultural incongruence operates not merely by presenting unexpected information, but by turning the adequacy of visitors’ existing cultural interpretations into a problem requiring further cognitive processing.

Study 1 further showed a significant indirect effect of the cultural incongruence manipulation on critical reflection through epistemic curiosity. This result suggests that reflection following cultural incongruence is not entirely automatic, but depends partly on whether participants develop an inquiry-oriented motivation to understand the cultural background and symbolic logic of the exhibit. Epistemic curiosity may sustain attention to the explanatory gap created by the conflict between initial judgment and actual cultural meaning, encouraging participants to seek further information and compare competing interpretations. Previous research similarly conceptualizes epistemic curiosity as a motivational state activated by perceived knowledge gaps and directed toward learning and exploratory behavior (Litman et al., 2005; Schmidt and Rotgans, 2021). During this process, the question of “what does this exhibit actually mean?” may extend to “why did I interpret it differently?”, bringing the cultural associations and implicit assumptions underlying the initial interpretation into the scope of reflection. Therefore, the significant indirect effect supports epistemic curiosity as a motivational pathway linking cultural incongruence with critical reflection.

Study 2 showed that critical reflection was positively associated with cultural perspective taking and that this relationship became stronger as the need for cognition increased. Critical reflection may help visitors recognize that their initial interpretations are shaped by culturally situated experiences and assumptions, but such recognition is not equivalent to understanding another cultural standpoint. Cultural perspective taking additionally requires visitors to consider the historical context, values, beliefs, and social functions represented by the exhibit. This result is consistent with transformative learning theory, which proposes that reassessing existing assumptions can create space for alternative perspectives (Mezirow, 1998; Taylor, 2007), and with evidence linking critical reflection to changes in cultural awareness and perspective understanding (Carlson et al., 2025). Because constructing an alternative cultural interpretation requires sustained cognitive effort, visitors with a stronger need for cognition may be more willing to process complex contextual information and compare competing explanations. Those with a weaker need for cognition may instead remain at the level of acknowledging that their initial interpretation was incomplete. This pattern is consistent with established associations between need for cognition, cognitive engagement, and learning performance (Lins de Holanda Coelho et al., 2020; Liu and Nesbit, 2024). In this respect, Study 2 complements the experimentally examined antecedent process in Study 1 by providing experience-based evidence for the downstream relationship between critical reflection and cultural perspective taking. The two studies therefore illuminate different stages of the proposed framework through different methodological approaches.

### 6.2 Theoretical implications

This study advances museum learning research by introducing cultural incongruence as a theoretically distinct feature of exhibit interpretation. Its significance lies not simply in presenting unfamiliar or surprising content, but in exposing the limits of an interpretation that initially appears culturally plausible. This shifts attention from what visitors know about an exhibit to how they recognize that their existing cultural frame is insufficient. In this sense, cultural incongruence adds a more precise account of how exhibit design can create conditions for deeper meaning-making and connects schema incongruity with the study of culturally situated interpretation in museums (Meyers-Levy and Tybout, 1989; Vogl et al., 2020; Berweger et al., 2023).

The study also elaborates on transformative learning theory by clarifying the motivational link between a disorienting experience and critical reflection. Transformative learning has long emphasized that challenges to existing meaning frameworks can prompt the reassessment of prior assumptions, yet the transition from perceived disruption to sustained reflection remains less clearly specified (Taylor, 2007; Holdo, 2023; McClain, 2024). By incorporating epistemic curiosity, this study suggests that such a transition involves more than noticing that an interpretation is inadequate. It also involves a motivation to resolve the explanatory gap and examine the basis of the original judgment. This places inquiry motivation within the reflective process and provides a more differentiated account of how culturally disorienting experiences may become educationally meaningful (Loewenstein, 1994; Schmidt and Rotgans, 2021).

The study further develops the cultural dimension of transformative learning by distinguishing the reconsideration of one’s assumptions from the construction of another cultural standpoint. Critical reflection may loosen reliance on familiar interpretive frames, whereas cultural perspective taking requires the additional integration of another culture’s values, history, and social meanings. Treating cultural perspective taking as a proximal learning outcome gives greater specificity to what reflective change may involve in museum education. The moderating role of need for cognition further indicates that this process is shaped by visitors’ willingness to engage with complex and effortful information, thereby adding an individual-difference boundary to explanations of how reflection supports culturally situated understanding (Mezirow, 1998; Wang et al., 2003; Lins de Holanda Coelho et al., 2020; Liu and Nesbit, 2024).

### 6.3 Practical implications

Museums can make more effective educational use of cultural incongruence by conducting deeper research into the cultural histories and interpretive layers of collection objects. Curatorial work should move beyond basic descriptions of form, material, provenance, or chronology to examine how an object’s appearance relates to its ritual functions, symbolic conventions, social roles, and historical uses. This deeper investigation can reveal meanings that are not readily inferable from observable features and may challenge interpretations based on familiar cultural schemas. Once identified, these less visible tensions can be incorporated into exhibition narratives so that the contrast between an apparently plausible first impression and a culturally grounded interpretation becomes a meaningful starting point for inquiry and reflection.

Moreover, museums should avoid reducing cultural incongruence to a surprising fact followed by a corrective explanation. Interpretive materials can guide visitors through the cultural logic that makes the unexpected meaning intelligible. Information about ritual practice, social function, historical circumstances, and symbolic systems can help visitors understand why an object carries meanings that differ from those suggested by its appearance. Reflective prompts can then direct attention to the basis of the initial judgment by asking which visual cues, prior experiences, or familiar associations shaped it. This combination of contextual explanation and reflective questioning can encourage visitors not only to acquire additional information but also to examine the assumptions through which they interpret culturally unfamiliar objects.

Interpretive design should further support the transition from reconsidering an initial judgment to engaging with the standpoint represented by the exhibit. This requires presenting objects in relation to the values, practices, historical experiences, and social relationships through which their meanings have been formed. Culturally grounded narratives, multiple interpretive voices, and carefully structured comparisons can help visitors understand how an object is situated within a broader system of meaning rather than treating its cultural significance as an isolated fact. Because visitors differ in their inclination to engage in effortful processing, museums can organize these resources at different levels of cognitive demand. Core explanations can establish the essential context, while extended narratives, comparative evidence, and guided reflection can provide opportunities for more sustained examination of alternative interpretations and their underlying assumptions.

### 6.4 Limitations and future research

Several limitations provide directions for future research. Study 1 used a fictional exhibit and an online scenario to provide a controlled test of the immediate effects of cultural incongruence. Although this design strengthened experimental control, it could not fully reproduce the sensory, spatial, and social features of an actual museum visit. The relatively homogeneous sample of Chinese university students also limits the generalizability of the findings to the broader museum-going public, whose responses may vary with age, educational background, and prior museum experience. In addition, although the two experimental conditions used the same visual object and presentation format, they differed in the ritual context and cultural meaning described in the exhibit label. These differences may have carried distinct affective connotations, making it difficult to isolate cultural incongruence completely from other features of the scenario. Future research could refine the manipulation by holding ritual context and affective valence more closely constant while varying cultural incongruence, and extend the experiment to authentic museum settings with more heterogeneous visitor samples.

Study 2 relied on retrospective self-reports collected at a single point in time. Although the critical-incident recall approach anchored participants’ responses to a specific museum or cultural-exhibition experience, the recalled exhibits and experiences varied across participants, and cultural incongruence and epistemic curiosity were not assessed within those experiences. The conditions preceding critical reflection in naturally occurring museum visits therefore remain uncertain. Moreover, the observed relationships among critical reflection, need for cognition, and cultural perspective taking do not establish temporal or causal ordering, and recall-based reports may be affected by memory reconstruction or by the salience of the experience selected. Prospective longitudinal, experience-sampling, or field-based designs could capture these psychological responses closer to the museum encounter and provide stronger evidence regarding how they develop over time.

Finally, the two studies examined distinct but theoretically connected parts of the proposed framework rather than the complete sequence within a single design. Study 1 provided experimental evidence for the relationships among cultural incongruence, epistemic curiosity, and critical reflection, whereas Study 2 examined the downstream association between critical reflection and cultural perspective taking and the moderating role of need for cognition in recalled museum experiences. Because the studies relied on independent samples, the evidence across these stages cannot be treated as a within-person test of the full process. The present findings should therefore be interpreted as complementary evidence for different parts of the proposed framework rather than as a direct test of the entire sequential process. Future research could test the full framework within the same participants and museum experience, with the proposed constructs assessed at multiple points during and after the visit.

## Data Availability

The raw data supporting the conclusions of this article will be made available by the authors, without undue reservation.
