# Supplementary material for: When cultural meanings defy expectations: cultural incongruence, epistemic curiosity, critical reflection, and cultural perspective taking in museum education
Source: Front Psychol. 2026 Sep 11;17:1942537. doi: 10.3389/fpsyg.2026.1942537 (PMC13648035; doi:10.3389/fpsyg.2026.1942537)
Supplement: Supplementary file 1 [file Supplementary_file_1.docx]

Supplementary Material

This supplementary material reports the complete adapted English wording of the measurement items used in the two studies. Where appropriate, the items were contextualized to the museum-exhibit setting. Chinese versions of these items were administered to participants following translation and independent back-translation.

*Unless otherwise noted, all items were rated on a 7-point Likert scale (1 = strongly disagree, 7 = strongly agree). Items marked (R) were reverse-scored.*

# 1 Supplementary Table S1. Study 1 measurement items

| **Construct** | **Item** | **Adapted English item** |
| --- | --- | --- |
| Epistemic curiosity | EC1 | I would like to explore the cultural meaning of this exhibit in greater depth. |
|  | EC2 | The cultural meaning of this exhibit is fascinating to me. |
|  | EC3 | I am curious to learn more about the culture behind this exhibit. |
|  | EC4 | I think it would be interesting to gain a deeper understanding of this exhibit. |
|  | EC5 | I want to know more about the cultural background of this exhibit. |
|  | EC6 | I am not curious about the cultural meaning of this exhibit. (R) |
| Critical reflection | CR1 | This exhibit made me reconsider some of my own assumptions. |
|  | CR2 | This exhibit challenged some ideas that I had previously taken for granted. |
|  | CR3 | This exhibit made me rethink how I usually interpret cultural objects. |
|  | CR4 | This exhibit made me realize that some of my previous beliefs may have been incomplete or mistaken. |
| Perceived cultural incongruence (manipulation check) | MC1 | The cultural meaning of this exhibit was different from what I initially expected. |
|  | MC2 | The meaning described in the exhibit label did not fit well with the object’s appearance. |
|  | MC3 | There was a clear mismatch between how the exhibit looked and what it meant in this culture. |

# 2 Supplementary Table S2. Study 2 measurement items

| **Construct** | **Item** | **Adapted English item** |
| --- | --- | --- |
| Critical reflection | CR1 | This exhibit made me reconsider some of my own assumptions. |
|  | CR2 | This exhibit challenged some ideas that I had previously taken for granted. |
|  | CR3 | This exhibit made me rethink how I usually interpret cultural objects. |
|  | CR4 | This exhibit made me realize that some of my previous beliefs may have been incomplete or mistaken. |
| Need for cognition | NC1 | I prefer complex problems to simple ones. |
|  | NC2 | I like being responsible for situations that require a lot of thinking. |
|  | NC3 | Thinking is not something I consider enjoyable. (R) |
|  | NC4 | I would rather do something that requires little thought than something that challenges my thinking. (R) |
|  | NC5 | I enjoy tasks that require me to find new solutions to problems. |
|  | NC6 | I prefer an important and intellectually challenging task to one that is easier but requires little thought. |
| Cultural perspective taking | CPT1 | This exhibit helped me see the object or practice from the perspective of the people whose culture it represented. |
|  | CPT2 | This exhibit helped me understand why people in that cultural context might interpret or use the object differently from how I would. |
|  | CPT3 | I tried to imagine what this exhibit might mean to the people culturally connected to it. |
|  | CPT4 | This exhibit helped me understand the cultural beliefs, values, or practices behind it. |
|  | CPT5 | I tried to understand this exhibit within its own cultural context rather than judging it only by my usual standards. |
|  | CPT6 | I found it difficult to understand the cultural viewpoint represented by this exhibit. (R) |
|  | CPT7 | Even after the visit, I mainly understood this exhibit according to my own familiar cultural assumptions. (R) |
